# Mass Spectrometry-Based Metabolomic and Lipidomic Analyses of the Effects of Dietary *Platycodon grandiflorum* on Liver and Serum of Obese Mice under a High-Fat Diet

**DOI:** 10.3390/nu9010071

**Published:** 2017-01-17

**Authors:** Hye Min Park, Kab-Tae Park, Edmond Changkyun Park, Seung II Kim, Myung Sook Choi, Kwang-Hyeon Liu, Choong Hwan Lee

**Affiliations:** 1 Department of Bioscience and Biotechnology, Konkuk University, 120 Neungdong-ro, Gwangjin-gu, Seoul 05029, Korea; ramgee@naver.com; 2 BK21 Plus KNU Multi-Omics Based Creative Drug Research Team, College of Pharmacy and Research Institute of Pharmaceutical Sciences, Kyungpook National University, Daegu 41566, Korea; pkt0531@gmail.com; 3 Division of Life Science, Korea Basic Science Institute, Daejeon 34133, Korea; edpark@kbsi.re.kr (E.C.P.); ksi@kbsi.re.kr (S.I.K.); 4 Center for Convergent Research of Emerging Virus Infection, Korea Research Institute of Chemical Technology, 141 Gajeong-ro, Yuseong-gu, Daejeon 34114, Korea; 5 Department of Food Science and Nutrition, Kyungpook National University, Daegu 41566, Korea; mschoi@knu.ac.kr

**Keywords:** amino acids, glycerophospholipids, high-fat diet, metabolite profiling, obesity, *Platycodon grandiflorum*

## Abstract

We aimed to identify metabolites involved in the anti-obesity effects of *Platycodon grandiflorum* (PG) in high-fat diet (HFD)-fed mice using mass spectrometry (MS)-based metabolomic techniques. C57BL/6J mice were divided into four groups: normal diet (ND)-fed mice, HFD-fed mice, HFD with 1% PG extract-fed mice (HPGL), and HFD with 5% PG extract-fed mice (HPGH). After 8 weeks, the HFD group gained more weight than the ND group, while dietary 5% PG extract attenuated this change. The partial least squares discriminant analysis (PLS-DA) score plots showed a clear distinction between experimental groups in serum and liver markers. We also identified 10 and 32 metabolites in the serum and liver, respectively, as potential biomarkers that could explain the effect of high-dose PG added to HFD-fed mice, which were strongly involved in amino acid metabolism (glycine, serine, threonine, methionine, glutamate, phenylalanine, ornithine, lysine, and tyrosine), TCA cycle (fumarate and succinate), lipid metabolism (linoleic and oleic acid methyl esters, oleamide, and cholesterol), purine/pyrimidine metabolism (uracil and hypoxanthine), carbohydrate metabolism (maltose), and glycerophospholipid metabolism (phosphatidylcholines, phosphatidylethanolamines, lysophosphatidylcholines, and lysophosphatidylethanolamines). We suggest that further studies on these metabolites could help us gain a better understanding of both HFD-induced obesity and the effects of PG.

## 1. Introduction

*Platycodon grandiflorum* (PG) is a perennial plant from the Campanulaceae family, well known as a traditional herbal medicine for the treatment of asthma, diabetes, and respiratory disorders. It contains diverse bioactive compounds such as triterpenoid saponins, flavonoids, polyphenols, and fibers [1,2,3]. PG also possesses antioxidant, anticancer, anti-inflammatory, and hepato-protective pproperties [4,5,6]. Furthermore, many researchers have reported on the anti-obesity effect of PG and its constituents through the reduction of total cholesterol (TC) and triglyceride (TG) levels, and the inhibition of pancreatic lipase activity [7,8,9].

Obesity is most commonly caused by a chronic imbalance between energy intake and energy expenditure [10]. Long-term high-fat intake induces weight gain and provokes changes in various biochemical parameters such as insulin, glucose, leptin, TC, and TG levels in the blood and liver [11,12]. Obesity can therefore be defined as a disorder characterized by an abnormal lipid metabolism. To understand the metabolic pathways and mechanisms involved in obesity more completely, high-throughput metabolomic analyses have recently been applied, using mass spectrometry (MS) and nuclear magnetic resonance (NMR) spectroscopy. Metabolomics, the analysis of a huge range of small molecules in a biological system, is playing an increasingly important role in evaluating endogenous metabolite alterations in tissues and biological fluids, discovering potential biomarkers for diseases such as diabetes and obesity, or developing therapeutic applications [13,14,15]. Amino acids, fatty acids, carnitine, acyl-carnitines, lysophosphatidylcholines (lysoPCs), and lysophosphatidylethanolamines (lysoPEs) have been established as biomarker candidates for obesity through metabolomic analyses in obese animal models [16,17,18]. Based on this information, untargeted metabolite profiling has been performed on biologically active products and single compounds used to treat obesity [19,20]. 

In this study, we investigated the anti-obesity effect of two different concentrations of PG extract in high-fat diet (HFD)-induced obese C57BL/6J mice by profiling endogenous and exogeneous metabolites in both the serum and liver. Analyses were performed using comprehensive mass spectroscopy (MS) instruments such as ultra-performance liquid chromatography (UPLC)-quadrupole time-of-flight (Q-TOF)-MS, gas chromatography (GC)-TOF-MS, and direct infusion-MS, combined with multivariate analyses, to identify metabolites that would enable a better understanding of the beneficial effect of PG in HFD-related obesity.

## 2. Materials and Methods

### 2.1. Chemicals and Reagents

Acetonitrile, water, dichloromethane, and methanol were purchased from Fisher Scientific (Pittsburgh, PA, USA) or Merck (Darmstadt, Germany). Methoxyamine hydrochloride, *N*-methyl-*N*-(trimethylsilyl) trifluoroacetamide (MSTFA), formic acid, pyridine, ammonium acetate, chloroform, methyl-*tert*-buthyl ether (MTBE), and triglycerides mixture standard (16:0/18:1/18:2) were purchased from Sigma Aldrich (St. Louis, MO, USA). Santa Cruz Biotechnology, Inc. (Dallas, TX, USA) supplied us with 2-chloro-l-phenylalanine. Phosphatidylcholine (PC) 18:0/16:0, phosphatidylethanolamine (PE) 16:0/18:1, lysoPC 18:0, and cholesteryl ester (CE) 16:0 were purchased from Avanti Polar Lipids (Alabaster, AL, USA). All chemicals and solvents were of analytical grade and commercially available.

### 2.2. Preparation of PG Extract

PG roots were obtained from Omniherb (Daegu, Korea). PG roots (10 kg) were extracted with 70% ethanol (10 volumes) at 50 °C for 6 h, concentrated under vacuum, and then lyophilized. The extract contained a variety of platycosides such as platycoside E, deapioplatycodin D3, platycodin D3, polygalacin D3, platyconic acid A, 3′′-*O*-acetylplatyconic acid A, platycodin D2, platycodin D, 3′′-*O*-acetylplatycodin D2, polygalacin D2, polygalacin D, 3′′-*O*-acetylplatycodin D, platycodin V, platycodin A, 2′′-*O*-acetylpolygalacin D2, and 2′′-*O*-acetylpolygalacin D [21].

### 2.3. Experimental Design

Male C57BL/6J mice (4 weeks old) were obtained from The Jackson Laboratory (Bar Harbor, ME, USA). All mice were kept in a room maintained at 24 °C with 12 h light/dark cycles, fed normal chow diet for 7 days and subsequently randomly divided into four groups as follows: the first group was fed a ND (16.58 kcal% fat, *n* = 8), the second group was fed a HFD (60 kcal% fat, *n* = 8), the third group fed a HFD plus 1% (*w*/*w*) PG (HPGL, *n* = 7), and the fourth group was fed a HFD plus 5% (*w*/*w*) PG (HPGH, *n* = 7), for 12 weeks. At the end of the experimental period, all mice were anesthetized with ether after a 12 h fast. Blood was collected and sent for analysis. The liver and fats were collected, weighed, and stored at −70 °C until analysis. All animal procedures were approved by the Korea Basic Science Institute (KBSI-AEC 1509).

### 2.4. Hepatic Morphology

Liver tissues were removed from the mice and subsequently fixed in a 10% neutral buffered formalin solution. Fixed tissues were embedded in paraffin, and 4-cm-thick sections were prepared and stained with hematoxylin and eosin (H&E). Stained areas were viewed using an optical microscope at 200× magnification.

### 2.5. Hepatic Biochemical Parameters in Serum

Aspartate aminotransferase (AST) and alanine aminotransferase (ALT) levels were measured using a commercial analytical system standardized according to the International Federation of Clinical Chemistry and Laboratory Medicine (IFCC) reference procedure. Total cholesterol (TC) was determined based on the cholesterol oxidase-HMMPS method.

### 2.6. Serum Extraction

To prepare serum extracts for GC-TOF-MS analysis, 100 µL of serum was mixed with 300 µL of ice-cold methanol containing 10 µL of 2-chloro-l-phenylalanine (0.2 mg/mL) as an internal standard, sonicated for 5 min, and then placed in a −20 °C freezer for 1 h. Samples were centrifuged at 4 °C and 13,500× *g* for 10 min. The supernatants were then filtered through 0.2 μm polytetrafluoroethylene (PTFE) filters, collected in new Eppendorf tubes and completely dried prior to GC-TOF-MS analysis. Dried serum extracts were oximated with 50 μL methoxyamine hydrochloride (20 mg/mL) in pyridine at 30 °C for 90 min, and then it was reacted with 50 μL of the derivatizing agent, *N*-methyl-*N*-trimethylsilyl-trifluoroacetamide (MSTFA), which was then incubated at 37 °C for 30 min.

To perform the serum lipid analysis, the serum was extracted using the Matyash method with some modifications [22,23]. In brief, 225 µL ice-cold MeOH was added to 10 µL serum, and the mixture vortexed for 10 s. Next, 750 µL ice-cold methyl tert-butyl ether (MTBE) was added along with 187.5 µL distilled water. After (14,000 *g* for 2 min, 4 °C), the supernatant was collected and completely dried. Prior to lipid profiling, the dried serum extract was reconstituted in 100 µL chloroform/methanol (1:9, *v*/*v*) and 5-fold diluted with the same containing 7.5 mM ammonium acetate.

### 2.7. Liver Tissue Extraction

The liver extracts were prepared following a modified version of Masson et al.’s method [24]. Frozen liver (150 mg) was added to mixture solvent along with MeOH and water (1:1, 1 mL) containing 10 µL 2-chloro-l-phenylalanine (0.5 mg/mL). The resulting solution was then homogenized (30 frequency/min) 3 times for 5 min using a mixer mill (MM400; Retsch^®^, Haan, Germany). The suspension was centrifuged at 4 °C and 11,250× *g* for 10 min, and the resulting supernatant (MeOH/water extracts, MW) was transferred into a 2 mL microcentrifuge tube. The remaining pellet was extracted again with 1 mL of the mixture solvent and the supernatant (dichloromethane/MeOH extracts, DM) was collected in a new microcentrifuge tube following centrifugation. Each extract solution was evaporated with a vacuum centrifuge. Dried MW and DM samples were stored at −80 °C until UPLC-Q-TOF-MS and GC-TOF-MS analyses. For the UPLC-Q-TOF-MS analysis, dried MW and DM samples were re-suspended with 700 µL methanol/water (1:1) and subsequently filtered through a 0.2-μm PTFE filter. For the GC-TOF-MS analysis, 100 µL aliquots of the MW and DM extracts dissolved in the methanol/water (1:1) solvent were evaporated with a vacuum centrifuge. Each dried MW and DM extract was reacted with derivatizing agents under the same conditions as those applied for the GC-TOF-MS analysis of serum extracts. 

For the hepatic lipids extraction, 400 µL ice-cold 75% methanol was added to 50 mg of liver tissue, which was subsequently completely homogenized. Next, 1 mL MTBE was added and the mixture was vortexed for 1 h at room temperature. Phase separation was then induced by adding 250 µL water and the mixture was incubated for 10 min at room temperature. After centrifugation (14,000× *g* for 15 min, 4 °C), 220 µL of supernatant was collected and completely dried prior to lipid profiling. Dried liver lipid extracts were reconstituted in 100 µL chloroform/methanol (1:9, *v*/*v*) and 10-fold diluted with the same containing 7.5 mM ammonium acetate.

### 2.8. GC-TOF-MS Analysis

An Agilent 7890A GC system equipped with an Agilent 7693 autosampler coupled to a Pegasus TOF-MS detector (Leco Corporation, St. Joseph, MI, USA) was used for the analyses. An Agilent HP-5MS capillary column with an internal diameter of 0.25 mm, a film thickness of 0.25 µm, and a length of 30 m was employed for the separation. Chromatographic-grade helium with a constant flow of 1.0 mL/min served as the carrier gas. The oven temperature was held at 75 °C for 2 min, increased to 300 °C at a rate of 15 °C/min, then maintained at 300 °C for 3 min. Mass data were collected in the electron impact (EI) mode set to 70 eV ionization energy and a full scan (*m*/*z* 50−600). The injector and transfer line temperatures were 250 and 240 °C, respectively. One microliter of reactant was injected into the GC-TOF-MS, without split for serum and DM liver extracts, or with split ratio 5:1 for MW liver extracts. A pooled quality control (QC) sample was analyzed at an interval of eight serum analytes.

### 2.9. UPLC-Q-TOF-MS Analysis

UPLC-Q-TOF-MS analysis was performed using a Waters Micromass Q-TOF Premier with UPLC Acquity system (Waters Corp., Milford, MS, USA) equipped with a UPLC mass spectrometer. The UPLC system was equipped with an Acquity UPLC BEH C18 column (100 × 2.1 mm, 1.7 μm particle size; Waters Corp.). The mobile phase consisted of 0.1% (*v*/*v*) formic acid in water (A) and 0.1% (*v*/*v*) formic acid in acetonitrile (B). The solvent gradient system consisted of the following conditions: held at 5% B for 1 min, gradually increased from 5% to 100% B for 9 min and held at 100% B for 1 min, then decreased to 5% B for 2 min, and maintained for 1 min. Five microliters of extract solution was injected, and the flow rate was maintained at 0.3 mL/min. An *m*/*z* of 100–1000 was designated with the electrospray ionization (ESI) in negative and positive modes. The operating parameters were as follows: ion source temperature, 80 °C; desolvation gas flow, 650 L/h; capillary voltage, 2.3 kV; cone voltage, 35 V. The V mode was used for the mass spectrometer and data were collected in the centroid mode with a scan accumulation time of 0.2 s. Leucine enkephalin was used as reference lock mass ([–] *m*/*z* 554.2615, [+] *m*/*z* 556.2771, 10 μL/min) by independent LockSpray interference. A pooled QC sample was analyzed at an interval of eight serum analytes.

### 2.10. Lipid Profiling by Direct Infusion MS

The lipid solution was infused into a Thermo linear ion trap quadrupole (LTQ) XL ion trap mass spectrometer (Thermo Fisher Scientific, West Palm Beach, FL, USA) using a chip-based nano-electrospray infusion system (TriVersa NanoMate, Advion Biosciences, Ithaca, NY, USA). The ionization voltage was set to 1.4 kV, gas pressure to 0.4 psi, and the source was controlled by Chipsoft software (version 8.3.1, Advion Biosciences, Ithaca, NY, USA). The capillary temperature was set to 200 °C and the tube voltage to 100 V. For lipid profiling, 10 µL of each sample was randomly loaded onto a 96-well plate of the TriVersa Nanomate ion source, to avoid bias in analysis. A pooled QC sample was loaded at the beginning, middle, and end of the batch. The 96-well plate was sealed with Thermowell aluminum sealing tape (Corning, The City of Corning, NY, USA) and then placed on the Nanomate cooling plate, which was set to 4 °C to prevent evaporation of the solvent. Full-scan spectra were collected at the *m*/*z* 400–1000 range in positive mode. The mass spectrum of each sample was acquired in profile mode over 2 min. A collision-induced dissociation (CID) was performed over an isolated width of 1.5 *m*/*z* units, with a 30 eV collision energy. All spectra were recorded using the Thermo Xcalibur software (version 2.1, Thermo Fisher Scientific, West Palm Beach, FL, USA).

### 2.11. Data Processing and Multivariate Analysis

GC-TOF-MS and UPLC-Q-TOF-MS data files were converted to CDF format using ChromaTOF v4.44 (Leco Co., St. Joseph, MI, USA) and MassLynx softwares (Waters Corp., Milford, MS, USA), respectively. After conversion, MS data were processed using the metAlign software package (http://www.metalign.nl) to obtain a data matrix containing retention times, accurate masses, and normalized peak intensities, using sample names and peak area information as variables. The resulting data matrix was processed using SIMCA-P+ (version 12.0, Umetrics, Umea, Sweden) for multivariate statistical analysis. For data processing of lipid profiles, nominal ion mass spectra data files from the ion trap mass spectrometer (“.raw” files) were directly loaded into the Genedata Expressionist MSX module (Genedata AG, Basel, Switzerland) [25]. Data on all detected peaks, including *m*/*z* and intensity values, were exported as Excel files. To normalize spectral data, the intensities of each sample were summed, and each value (-fold) was divided by the sum of the intensities. The resulting Excel data were exported to SIMCA P+ software (version 13.0, Umetrics, Umea, Sweden).

Principal component analysis (PCA) and partial least squares discriminant analysis (PLS-DA) modeling were performed to obtain information on differences in metabolite profiles between experimental groups. The discriminated variables were selected based on variable importance in the projection (VIP) value (>1.0 or >0.7) and *p*-value (<0.05, one-way ANOVA followed by Duncan’s multiple range test) using SIMCA-P+ software and PASW Statistics 18 (SPSS Inc., Chicago, IL, USA). Following multivariate statistical analysis, the peaks corresponding to selected variables were confirmed in the original chromatograms and were positively or tentatively identified using either commercial standard compounds in comparison with the mass spectra and retention time or on the basis of the NIST mass spectral database (National Institute of Standards and Technology, FairCom, Gaithersburg, MD, USA), in-house library, and references for GC-TOF-MS. For UPLC-Q-TOF-MS, the assignment of metabolites contributing to the observed variance was performed by elemental composition analysis software with the calculated mass, mass tolerance (mDa and ppm), double bond equivalents (DBEs), and iFit algorithm implemented in MassLynx and by the Human Metabolome Database (HMDB, http://www.hmdb.com) and Lipid Maps Database (http://www.lipidmaps.org). In addition, serum and liver lipids were identified by comparison with the MS/MS fragmentation patterns of commercially available standards or by using the LIPID MAPS Lipidomics Gateway (http://www.lipidmaps.org), the Human Metabolome Database (HMDB; http://www.hmdb.com), and/or our in-house lipid library (LipidBlast) [26]. Significantly different metabolites were represented as a fold change that was calculated by dividing the mean of the peak intensity of each metabolite from each of the two groups. The statistical significance in each metabolite between each of the two groups was determined by Student’s *t*-test (*p*-value < 0.05). In addition, the statistical analysis for mice characteristics and biochemical parameters variations between each group was evaluated using a one-way analysis of variance (ANOVA) followed by Duncan’s multiple range test.

## 3. Results

### 3.1. Effects of PG on HFD-Induced Obesity in C57BL/6J Mice

The average initial body weight of the C57BL/6J mice was 20.4 ± 1.2 g at the start of the experiment. The average weight of each group at the end of 12 weeks was 31 ± 4 g in the ND group, 49 ± 1 g in the HFD group, 45 ± 3 g in the HPGL group, and 32 ± 3 g in the HPGH group (Figure S1). Additionally, long-term high-fat feeding induced excess weight gains of liver, subcutaneous fat, and visceral fat, but 5% PG extract significantly reduced this effect (Table 1). Similar to what has been previously reported [21], however, there was no difference in food intake of mice fed a ND, HFD, HPGL, and HPGH.

The HFD induced significant changes in biochemical serum parameters such as increases in AST, ALT, and TC levels. These impacts were clearly attenuated with the addition of 5% PG extract, but not with 1% PG extract (Table 2).

**Figure 1:**
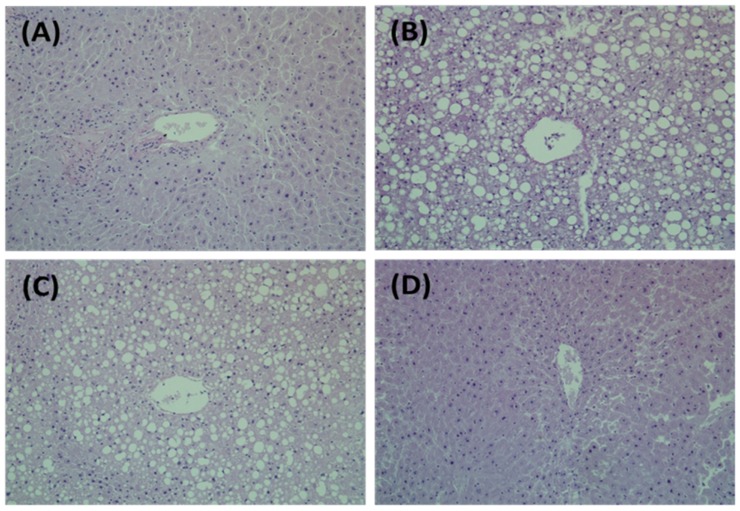
Effects of *Platycodon grandiflorum* (PG) extracts on Hepatic Morphology in a High-fat Diet-fed C57BL-6J. (**A**) ND, normal diet; (**B**) HFD, high-fat diet; (**C**) HPGL, high-fat diet with 1% *P. grandiflorum*; (**D**) HPGH, high-fat diet with 5% *P. grandiflorum*. Liver tissues were stained with hematoxylin and eosin (H&E). The stained slices were examined under an optical microscope at a 200-fold magnification.

Representative micrographs of liver tissues of mice stained with H&E are shown in Figure 1. ND mice had no lipid deposition in the liver and exhibited normal tissue morphologies. Fatty livers were histologically apparent in HFD mice, whereas the size and number of fat droplets considerably declined in liver tissues of HPGH mice.

### 3.2. Serum Metabolite Changes in HFD and PG Extract-Fed Mice

GC-TOF-MS analysis of mouse serum detected 32,969 variables which were subsequently applied to PCA and PLS-DA models to select the discriminated variables between the four experimental groups, based on a model with R2Xcum values, R2Ycum values, and Q2cum (Figure S2). The 3 dimensional PLS-DA score plot showed a clear discrimination between each group (Figure S2B). The scores of the first PLS component (PLS1), accounting for the greatest possible variance in the data set, and the following component (PLS2) were 12.7% and 5.2%, respectively. To select the major variables between the experimental groups, both VIP 1 and VIP 2 values (>1.0) resulting from PLS1 and 2, as well as *p*-values (<0.05) were used. Twenty-two variables were tentatively identified as biomarker candidates for the diagnosis of obesity and the anti-obesity effect of PG extract, distributed as follows: six amino acids, three organic compounds, eight carbohydrates, four fatty acids, and cholesterol (Table S1). Most serum metabolites were more affected by HFD feeding than PG extract treatment. The levels of amino acids and carbohydrates significantly increased under a HFD while those of organic compounds and fatty acids significantly decreased. Among them, the levels of ornithine and tryptophan in the HFD group were increased compared to the ND group, while those in PG treatment groups decreased compared to the HFD group in a PG dose-dependent manner (Table 3).

PCA and PLS-DA models were performed on the lipid profiles of mice sera using normalized values of only identified lipid structures (Figure S2), which included 158 lipids (9 cholesterol esters (CEs), 7 diacylglycerols (DG), 21 lysoPCs, 40 PCs, 6 PEs, and 75 triacylglycerols (TGs)) in the positive ion mode. All four groups (ND, HFD, HPGL, and HPGH) significantly diverged from each other as shown in the 3D PLS-DA score plot (Figure S2D). We chose a VIP cut-off value of 0.7 and a *p*-value of 0.05. Among the identified lipids, 25 metabolites, 2 CEs, 2 lysoPCs, 9 PCs, and 12 TGs, were affected by HFD feeding. The levels of TGs having higher double bonds (≥3) and of lysoPC 18:2 were significantly decreased by HFD. However, the levels of CEs with C20:3 and C20:4 as well as of lysoPC with C20:4 were significantly increased by HFD. Regarding the PCs, the HFD induced both an increase in PCs with long acyl chains (≥36) and a decrease in PCs with short acyl chains (≤34). Among them, PCs with C36:4, C38:3, and C38:4, as well as lysoPC 20:4 were identified as potential biomarkers to explain the effects of PG extract on HFD-induced obesity. The HFD-induced alterations to these metabolites were attenuated with 5% PG extract treatment. In addition, the levels of these four metabolites in the HPGH group did not differ from those detected in the in the ND group.

From these data, we figured out 10 potential serum biomarkers closely involved in the effect of 5% PG extract feeding on HFD-fed mice (Table 3).

### 3.3. Hepatic Metabolite Changes in HFD and PG Extract-Fed Mice

**Figure 2:**
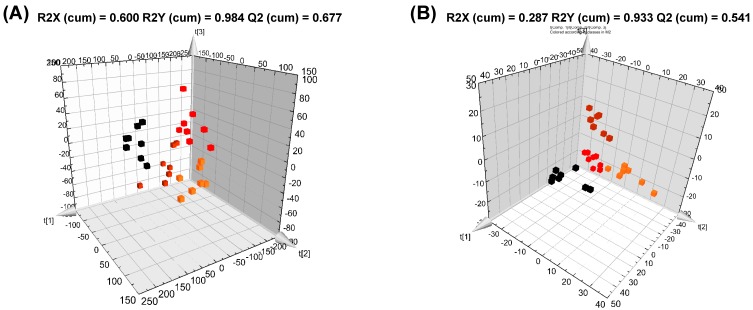

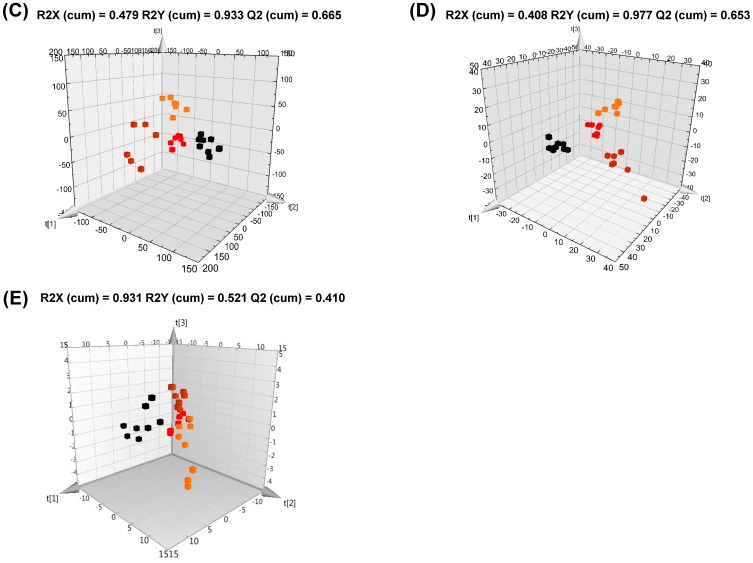
Three dimensional (3D) PLS-DA score plots derived from GC-TOF-MS (**A**,**C**) and UPLC-Q-TOF-MS (**B**,**D**) data sets in negative ion mode for MW (**A**,**B**) and DM (**C**,**D**) extracts, and direct infusion nanoelectrospray-MS data set (**E**) of liver in mice fed a High-Fat Diet (HFD) including *Platycodon grandiflorum*. Black square ND, normal diet; red square HFD, high-fat diet; orange square HPGL, high-fat diet with 1% *P. grandiflorum*; brown square HPGH, high-fat diet with 5% *P. grandiflorum*.

GC-TOF-MS and UPLC-Q-TOF-MS analyses of liver tissues detected 25,846 and 25,258 variables, respectively, while UPLC-Q-TOF-MS analysis detected 2348 and 3929 variables in MW and DM extracts, respectively. These variables were applied to PCA and PLS-DA models. In both GC-TOF-MS and UPLC-Q-TOF-MS analyses (Figure S3), the PCA score plots derived from the MW extract showed a more defined distinction between the four groups than those obtained from the DM extract. According to the PLS-DA score plots (Figure 2), the ND, HFD, HPGL, and HPGH groups were all clearly discriminated from each other by PLS1 and PLS2, based on the model applying R2Xcum and R2Ycum values of 0.287–0.600 and 0.933–0.977, respectively, and with Q2 cum values of 0.541–0.777. The scores of PLS1 and PLS2 in the MW extract were 23.3% and 11.1%, respectively, from the GC-TOF-MS data set, while they were 10.5% and 8.7%, respectively, from the UPLC-Q-TOF-MS data set. In the DM extract, the corresponding values were 16.8% and 10.6% in the GC data set, and 8.8% and 5.6% in the UPLC data set. The variables significantly contributing to the discrimination between the experimental groups were selected based on the VIP value (>1.0) and *p*-value (<0.05).

Moreover, we identified 136 lipids including 2 DGs, 9 lysoPCs, 30 PCs, 28 PEs, and 67 TGs through direct infusion MS analysis. PCA and PLS-DA models were performed using the normalized data of these lipids. The 3D PLS-DA score plots showed more defined discriminations between the groups than PCA (Figure 2 and Figure S2) and were validated with fitness R2Xcum and R2Ycum values of 0.931 and 0.521, respectively, and with a predictability Q2 cum value of 0.410. The scores of PLS1 and PLS2 in the lipid extract were 49% and 40%, respectively. Major metabolites were selected on the basis of both a VIP value above 0.7 and a *p*-value below 0.05. 

Sixty-two metabolites were identified from GC-TOF-MS data sets in both MW and DM extracts of liver tissues, which fell into six categories including amino acids, organic and inorganic compounds, lipids, carbohydrates, and nucleobases (Table S3).

Fifteen amino acids—alanine, valine, isoleucine, proline, glycine, serine, threonine, aspartic acid, methionine, pyroglutamate, glutamate, phenylalanine, ornithine, lysine, and tyrosine—showed a significant decrease under a HFD. However, their levels showed a dose-dependent trend toward recovery with PG treatment. In particular, the levels of eight amino acids including serine, threonine, methionine, glutamic acid, phenylalanine, ornithine, lysine, and tyrosine were increased compared to the HFD group in a PG dose-dependent manner (Table 4).

We also detected a decrease in succinic and fumaric acids, known as intermediates in the citric acid cycle, under a HFD, which was significantly attenuated by 5% PG treatment. Regarding lipids, the HFD induced both a decrease in fatty acid and cholesterol levels and an increase in fatty acid methyl ester contents. However, some fatty acids such as stearic, oleic, and docosahexaenoic acids showed a recovery pattern with the addition of 1% PG, while other fatty acid methyl esters including linoleic and oleic acid methyl esters showed attenuation of HFD-induced changes with the addition of 5% PG. The cholesterol levels in both the HPGL and HPGH groups appeared to return to their original normal values.

Together with the lipids, four metabolites related to pyrimidine and purine metabolisms—uracil, uridine, inosine, and hypoxanthine—decreased 0.33 to 0.57-fold in the HFD group as compared to the ND group. However, the uracil and hypoxanthine contents significantly increased with 5% PG feeding. We also identified numerous carbohydrates including sugars and sugar alcohols. Except for five metabolites (glycerol, arabitol, galacturonic acid derivative, myo-inositol, and one saccharide), most of the metabolites’ levels increased with HFD feeding. More particularly, lactose and maltose in the HFD group showed dramatic increases, 19.19 and 12.33-fold compared to the ND group, respectively. In the HPGL group, which showed the lowest anti-obesity activity, these sugars’ levels were lower than in the HPGH group.

From the UPLC-Q-TOF-MS data set, 25 metabolites were tentatively identified as discriminated variables explaining the effects of the HFD and PG feeding (Table S4). Most of the metabolites detected in MW and DM extracts from liver were significantly modulated by HFD. The levels of two amino acids (lysine and histidine), and two bile acids, decreased. In particular, changes in lysophospholipid, lysoPC, and lysoPE levels were significant. LysoPEs including C16:0, C18:0, C18:1, two forms (sn-1 and sn-2) of C18:2, and C22:6, decreased under a HFD. Some lysoPCs, such as C16:0, C16:1, and two types of C18:2 also decreased, while two types of C18:1, C20:3, C20:4, and C22:6 increased under a HFD. Overall, these lysophospholipids showed significant attenuations with 1% and 5% PG extract treatments.

From the hepatic lipids results, we confirmed the existence of significant changes in the levels of 28 hepatic metabolites (one lysoPC, 4 PCs, 3 PEs, and 20 TGs) in HFD-induced obese mice (Table S5). In particular, the levels of TGs with higher double bond numbers (>3) were significantly lower in the HFD group, while those of TGs with lower double bond numbers (≤3) were higher. Additionally, the levels of PEs, PCs with short acyl chains (≤34), and lysoPC with C18:2 significantly decreased after HFD feeding, whereas the level of PC with 38:9 increased. After 5% PG treatment, the levels of four particular metabolites (PE 36:2, PE 36:3, PC 38:9, and lysoPC 18:2) in liver tissues recovered to the level of the mice fed the normal diet.

Based on these data, 30 metabolites were identified as potential hepatic biomarkers explaining the impact on a high dose of PG extract intake in liver tissues from HFD-fed mice (Table 4).

## 4. Discussion

In this study, PG reduced the extent and features of obesity. Clinical characteristic changes induced by the HFD in the serum, liver, and fat were significantly attenuated through supplementation with PG extract. In particular, the inhibitory effect on obesity of high-dose (5%) PG treatment was superior to that of low-dose (1%). Additionally, we revealed the anti-obesity effect of PG on serum and liver metabolites using MS-based metabolomic techniques. A high-fat diet supplemented with PG extract influenced hepatic metabolites more than serum metabolites. We also identified a total of 43 and 112 metabolites in serum and liver that were closely associated with the HFD and/or PG extract feeding, respectively. Among them, 10 and 32 metabolites were revealed to be potential biomarkers in response to a high-dose treatment of PG in serum and liver from HFD-fed mice (Table 3 and Table 4). In serum, four amino acids, three organic compounds, eight carbohydrates, four fatty acids, two LysoPCs, two CEs, seven PCs, and 11 TGs were altered by HFD, while only seven metabolites (ornithine, tryptophan, one saccharide, LysoPC 20:4, PC 36:4 (16:0/20:4), PC 38:3 (18:0/20:3) and PC38:4 (18:0/20:4)) were significantly alleviated in the HPGH group, showing an anti-obesity effect. Lai et al. [27] recently reported on the alteration of serum metabolites in non-alcoholic fatty liver disease-induced C57BL/6J mice relative to HFD-fed mice. After 12 weeks of HFD feeding, amino acids including methionine, tryptophan, lysine, and glutamic acid, that are predominantly associated with oxidation, inflammation, collagen synthesis, and insulin secretion, showed significant increases. Shearer et al. [16] demonstrated that changes in serum metabolites such as an increased level of citrate, and decreased levels of glycine, lysine, leucine, suberate, and acetate, were involved in the energy metabolism of C57BL/6J mice with obesity and insulin resistance caused by the HFD for 12 weeks. From these results, we suggest that PG treatment might partially improve the impaired amino acid metabolism induced by high-fat feeding and provide an anti-obesity effect.

Ninety-five primary hepatic metabolites were significantly altered by HFD. With the exception of three fatty acid methyl esters, oleamide, nine carbohydrates, seven lysoPCs with C18:1, C20:3, C20:4 and C22:6, one PC 38:9, and nine TGs with C50:1, C50:2, C51:2, C52:1, C52:2, C54:1, C54:2, C54:3, and C56:3, most metabolites showed significantly decreased levels in the HFD group compared to the ND group. In particular, we observed that the levels of 16 amino acids had almost dropped by half in the liver of obese mice after 12 weeks of high-fat feeding, while some amino acids present within the serum showed the opposite trend. These data were partly concordant with a previous study [18]. Since the liver is the major organ involved in amino acid metabolism, it is largely responsible for maintaining amino acid homeostasis. Abnormal changes in amino acids have been shown to cause regulatory dysfunctions, potentially impacting energy metabolism, protein, fatty acid and urea synthesis, proteolysis, and cell signaling [28,29]. The levels of nine amino acids including glycine, serine, threonine, methionine, glutamate, phenylalanine, ornithine, lysine, and tyrosine, were reduced with HFD, and then increased through high-dose treatment with PG, indicating that PG extract might control and normalize abnormal amino acid metabolisms generated by HFD.

Our data showed that the concentrations of lactate, urea, succinic acid, fumaric acid, taurine, malonic acid, and gluconic acid were reduced after chronic high-fat diet feeding. The HFD induces impaired insulin signaling, resulting in elevated in vivo gluconeogenesis and oxidative TCA cycle flux. The induction of the TCA cycle function also generates hepatic oxidative stress and inflammation [30]. Additionally, the TCA cycle is critically linked to the urea cycle, which is responsible for ammonia detoxification and nitrogen excretion [31]. Within the urea cycle, the enzymes involved, such as carbamoyl phosphate synthase 1 (CPS1), ornithine transcarbamoylase (OTC), argininosuccinate synthase (ASS), argininosuccinate lyase (ASL), and arginase 1 (ARG1), were down-regulated by the HFD [32]. The decrease in levels of urea and ornithine, an intermediate of the urea cycle, shown by our data, may be based on the alteration of these key enzymes. TCA and urea cycles in the liver are closely related to amino acid levels, especially glutamate and aspartate [31,33,34]. During catabolism of glutamate family molecules, glutamine, arginine, ornithine, proline, and histidine are first converted into glutamate, and then transformed into α-ketoglutarate, a key intermediate in the TCA cycle. As mentioned above, proline, ornithine, glutamate, histidine, and aspartate showed reduced levels in the HFD group, but partially recovered with PG feeding. These results suggest that the HFD affects the intermediates of the TCA and urea cycles and that PG regulates them, together with amino acids.

Glutamate is also known to affect carbohydrate metabolism, particularly gluconeogenesis, which is the process of new glucose production in the liver and kidneys. The synthesized glucose can then be used for energy production by these tissues. The primary carbon skeletons used for gluconeogenesis are derived from pyruvate, lactate, glycerol, and the amino acids alanine and glutamine [33,35]. These substrates’ levels were significantly lower after the HFD, and dietary PG did not induce any recovery effect. Furthermore, hepatic glucose contents showed no differences between the ND and HFD groups. However, lactose and maltose levels increased more rapidly (19- to 12-fold, respectively) under a HFD than other carbohydrates, and were reduced in the HPGL group. A previous study reported a decrease in glucose levels and an increase in maltose levels in livers of HFD-induced obese mice, demonstrating that elevated insulin resistance generated by obesity is positively associated with hepatic contents of mono and di-saccharides, but not glucose, in obese mice [18]. Although the saccharides’ levels, altered by the HFD, significantly recovered following treatment with a low concentration of dietary PG extract, the HPGL group did not show any anti-obesity effect. At this stage, the mechanisms governing this phenomenon are still unknown. Therefore, further studies on the relation between anti-obesity effects and saccharide level alteration by low concentration dietary PG will be required.

We also identified variations in the levels of some metabolites related to purine and pyrimidine metabolism, such as uracil, uridine, inosine, hypoxanthine, and xanthine. Inosine is normally converted to hypoxanthine by purine nucleoside phosphorylase and then further converted into xanthine by xanthine oxidase. Uridine-containing uracil and ribose protect the liver against drug-induced hepatotoxicity and psychiatric disorders [36]. HFD intake led to the elevation or suppression of these hepatic nucleobase levels, indicating either oxidative stress promoting purine catabolism or glycogenesis dysfunction [37]. Moreover, we observed similar results to those previously obtained regarding the depletion of hepatic uracil, inosine, xanthine, and hypoxanthine. The groups treated with PG showed recovery patterns for these metabolites. From these results, it appears that the HFD induces alterations in the metabolisms of purines and pyrimidines, which can be attenuated in the presence of PG. 

Most importantly, in this study, we observed obvious changes in the levels of a variety of lipids. Among these, fatty acids with C18 and a double bond such as oleic (C18:1), linoleic (C18:2) and λ-linolenic (C18:3) acids showed level changes below 0.5-fold, while their methyl esters, including oleic and linoleic acid methyl esters, which were only detected in liver DM extracts, showed significant increases following HFD feeding. The dysregulation of hepatic lipid synthesis has been closely associated with obesity. Oosterveer et al. reported that the HFD promotes hepatic lipid accumulation and leads to the increased expression of lipogenic and cholesterogenic genes compared to chow-fed mice [38]. Additionally, another study described increases in unsaturated fatty acids (UFA) and monounsaturated fatty acids (MUFA) levels in conjunction with decreased levels of saturated fatty acids (SFA) and polyunsaturated fatty acids (PUFA) in the liver of rats fed the HFD using both NMR and GC-FID/MS, which indicated enhanced peroxidation of PUFA and oxidative stress [37]. When a high dose of PG extract was provided to HFD-fed mice, these fatty acids’ levels generally recovered. Noh et al. reported that the action of hepatic cytosol fatty acid synthase (FAS), as a representative enzyme mediating lipid biogenesis, and the expression of stearoyl-CoA desaturase 1 (SCD1), which is related to the synthesis and regulation of unsaturated fatty acids, were significantly reduced in C57BL/6 mice treated with the HFD plus total extract of PG, compared with the HFD group [39]. Based on these previous reports, PG may influence hepatic fatty acid content by regulating FAS and SCD1 activities in HFD-induced obese mice. In particular, our previous study suggested that PG markedly decreased the hepatic TG and cholesterol content, as well as lipogenic mRNA expression (FAS, SCD1), which contributed to the decreased hepatic lipid droplet formation and lipid accumulation [21].

We observed significant increases in the levels of PCs with long acyl chains (≥36) in serum and liver samples in conjunction with significant decreases in the levels of PCs with short acyl chains (≤34) following HFD feeding. The levels of hepatic PEs were also significantly lower within the HFD group. LysoPCs and LysoPEs, which are derived from PCs and PEs, respectively, showed alterations in their levels dependent on fatty acyl chain length and the number of double bonds. Levels of lysoPCs containing unsaturated acyl chains (≥18) increased in the HFD group as compared to the ND group, while the levels of those containing saturated or monounsaturated acyl chains decreased. Nam et al. recently reported that the levels of PCs with relatively long acyl groups were high, while the levels of PCs with short acyl groups were relatively low in HFD-obesity prone groups compared with HFD-obesity resistant mice [40]. Our findings are also supported by previous works, which showed the down-regulation of PEs with C34:2, C36:2, and C36:3 in livers of HFD-fed mice [41,42].

In general, the biosynthetic pathways of PCs and PEs are regulated by SCD1, which modulates the glycerophospholipid profile [43]. PCs are mainly synthesized through, firstly, the cytidine diphosphate (CDP)-choline pathway, which regroups medium chain species (e.g., 16:0/18:0) [44], and secondly, through the PE *N*-methyltransferase (PEMT) pathway, consisting of species with longer chains (e.g., 18:0/20:4) [45]. The CDP-choline pathway of PCs synthesis was up-regulated, whereas the PEMT pathway was down-regulated in the liver of SCD1−/− mice [43]. A previous study reported that mice lacking PEMT were protected against HFD-induced obesity and insulin resistance, confirming the relationship between the down-regulation of PEMT and anti-obesity effects [45]. In this study, the decline in levels of serum PCs with relatively long acyl chains (≥36), as well as the elevation in hepatic levels of PEs with long acyl chains and PCs with short acyl chains (≤34), was detected in the HFD plus 5% PG extract-fed mice. Furthermore, altered lysoPC and lysoPE levels following HFD feeding also recovered through PG treatment. Several researchers reported that the administration of PG extracts to obese mice reactivates AMP-activated protein kinase (AMPK) phosphorylation [46,47], which induces nitric oxide production through the activation of endothelial nitric oxide synthase [48]. Nitric oxide is partially associated with the biosynthesis of PCs via the PEMT pathway [49]. Additionally, many studies have suggested that the changes in lysoPCs levels might be responsible for the emergence of metabolic disorders associated with oxidative stress and inflammation [18,50]. Although the relationship between anti-obesity effects and phospholipids was not clearly detected in the present study, based on our findings, PG might have an inhibitory effect on obesity through its regulation of phospholipid synthesis.

The changes in TG levels showed different patterns depending on the number of double bonds. Similar observations were made in our previous report, which showed a significant decrease in levels of TGs with higher double bond numbers (≥3) in the serum of HFD-fed mice [25]. In addition, Nam et al. recently reported that the levels of TGs with higher degrees of unsaturation (>4) decrease in plasma of HFD-obesity prone mice compared to those measured in HFD-obesity resistant mice, while levels of TGs with lower degrees of unsaturation (<4) increased [40]. Valenzuela et al. recently reported that a diminution in liver Δ5- and Δ6-desaturase activities, as well as a depletion of polyunsaturated fatty acids were induced by the HFD [51]. However, PG treatment did not generate clear recovery patterns of TGs levels, which had been significantly altered within the HFD group, compared to those of the ND group. 

**Figure 3:**
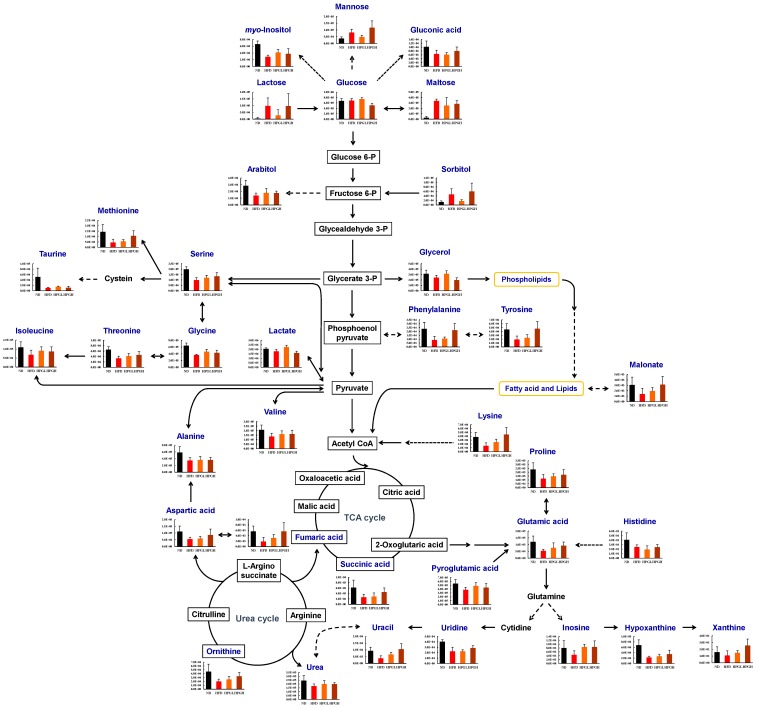
Proposed metabolic pathway derived from the metabolites significantly altered in liver tissues depending on the administration of *Platycodon grandiflorum* to High-Fat Diet (HFD)-induced obese mice. In the histograms, the peak intensity was plotted on the y-axis, while the different experimental groups were plotted on the *x*-axis. Black square ND, group fed with a normal diet; red square HFD, group fed with a high-fat diet; orange square HPGL, group fed with a high-fat diet and 1% *P. grandiflorum*; brown square HPGH, group fed with a high-fat diet and 5% *P. grandiflorum*.

Considering the alteration of several metabolite levels in serum and liver samples of HFD-induced obese mice observed using combined MS analyses, we suggest a metabolic pathway describing a number of metabolites as potential biomarkers of anti-obesity effects (Figure 3). However, further studies on the molecular mechanisms influencing the relationship between altered serum and hepatic metabolites and PG treatment are needed.

## 5. Conclusions

In the present study, we confirmed the anti-obesity effect and the changes in serum and hepatic metabolites upon PG treatment in HFD-fed mice. More specifically, high-dose supplementation (5%) of PG generated remarkable alterations in the levels of several metabolites, including amino acids, PCs, PEs, lysoPCs, and lysoPEs, and showed an inhibitory effect on obesity through the regulation of metabolic pathways intimately associated with these metabolites. These metabolites may serve as valuable biomarkers toward understanding the mechanisms through which the consumption of PG-containing diets can help treat or prevent obesity.

## Figures and Tables

**Table 1:** Characteristics of mice fed a Normal Diet (ND), a High-Fat Diet (HFD), HFD with 1% *Platycodon grandiflorum* (PG) extract (HPGL), and HFD with 5% PG extract (HPGH).

Weight (g)	ND	HFD	HPGL	HPGH
Body	31 ± 4 ^a^	49 ± 1 ^b^	43 ± 3 ^b^	32 ± 3 ^a^
Liver	1 ± 0.2 ^a^	2 ± 0.9 ^b^	2 ± 0.1 ^b^	1 ± 0.1 ^a^
Subcutaneous fat	1 ± 0.4 ^a^	5 ± 2 ^b^	5 ± 0.6 ^b^	2 ± 1 ^a^
Visceral fat	1 ± 0.4 ^a^	3 ± 0.7 ^c^	4 ± 0.4 ^c^	2 ± 1 ^b^

The data were evaluated using one-way analysis of variance (ANOVA) followed by Duncan’s multiple range test. Means with different letters, e.g., “a” or “b” are statistically different. Differences were considered significant at *p* values < 0.05.

**Table 2:** Biochemical serum parameters of mice fed a Normal Diet (ND), a High-Fat Diet (HFD), HFD with 1% *Platycodon grandiflorum* (PG) extract (HPGL), and HFD with 5% PG extract (HPGH).

Parameters	ND	HFD	HPGL	HPGH
AST (U/L)	159 ± 90 ^a^	254 ± 96 ^b^	199 ± 70 ^a,b^	175 ± 50 ^a,b^
ALT (U/L)	39 ± 19 ^a^	201 ± 133 ^b^	187 ± 92 ^b^	35 ± 20 ^a^
Total cholesterol (mg/dL)	141 ± 18 ^a^	264 ± 62 ^b^	254 ± 25 ^b^	195 ± 51 ^c^

The data were evaluated using one-way ANOVA followed by Duncan’s multiple range test. Means with different letters, e.g., “a” or “b” are statistically different. Differences were considered significant at *p* values < 0.05.

**Table 3:** List of potential serum biomarkers in mice fed a High-Fat Diet (HFD) including 5% *Platycodon grandiflorum* using various MS analyses.

No.	Metabolite	Fold Change ^1^		
		HFD/ND	HPGL/HFD	HPGH/HFD
1	Ornithine	1.38 ^#^	0.76 ^#^	0.72 ^#^
2	Tryptophan	1.26 ^#^	0.85 ^#^	0.78 ^#^
3	Saccharide *	1.67 ^#^	0.92	0.79 ^#^
4	LysoPC 20:4	1.65 ^#^	0.98	0.66 ^#^
5	PC 34:2 (16:0/18:2)	0.55 ^#^	0.88	1.39 ^#^
6	PC 36:4 (16:0/20:4)	1.50 ^#^	1.11	0.71 ^#^
7	PC 38:4 (18:0/20:4)	2.28 ^#^	1.01	0.60 ^#^
8	PC 38:3 (18:0/20:3)	2.07 ^#^	1.00	0.65 ^#^
9	PC 38:3	1.29 ^#^	1.07	0.77 ^#^
10	TG 52:3	0.53 ^#^	0.66	2.06 ^#^

ND, group fed with a normal diet; HFD, group fed with a high-fat diet; HPGL, group fed with a high-fat diet and 1% *P. grandiflorum*; HPGH, group fed with a high-fat diet and 5% *P. grandiflorum*. ^1^ Fold change was calculated by dividing the mean of the peak intensity of each metabolite from each of the two groups. * Saccharide was not successfully identified, but its mass fragments were similar to general mass fragments of saccharides in GC-TOF-MS analysis. ^#^ Metabolites showing significant differences (*p* < 0.05) between groups as determined by Student’s *t*-test.

**Table 4:** List of potential hepatic biomarkers in mice fed a High-Fat Diet (HFD) including 5% *Platycodon grandiflorum* using various MS analyses.

No.	Metabolite	Fold Change ^1^		
		HFD/ND	HPGL/HFD	HPGH/HFD
1	Glycine	0.57 ^#^	1.27 ^#^	1.18 ^#^
2	Serine	0.50 ^#^	1.21	1.34 ^#^
3	Threonine	0.51 ^#^	1.25	1.37 ^#^
4	Methionine ^a^	0.31 ^#^	1.25	2.38 ^#^
5	Glutamic acid	0.45 ^#^	1.42	1.71 ^#^
6	Phenylalanine ^a^	0.39 ^#^	1.19	2.40 ^#^
7	Ornithine	0.44 ^#^	1.26	1.67 ^#^
8	Lysine ^a^	0.39 ^#^	1.63 ^#^	2.98 ^#^
9	Tyrosine ^a^	0.42 ^#^	1.24	2.47 ^#^
10	Urea	0.71 ^#^	1.15	1.15 ^#^
11	Succinic acid	0.42 ^#^	1.11	1.75 ^#^
12	Fumaric acid ^a^	0.34 ^#^	1.67	2.96 ^#^
13	Linolelidic acid methyl ester ^a^	1.53 ^#^	0.85 ^#^	0.54 ^#^
14	Oleic acid methyl ester ^a^	2.45 ^#^	1.56 ^#^	0.54 ^#^
15	Oleamide ^a^	1.21 ^#^	0.90	0.72 ^#^
16	Cholesterol	0.24 ^#^	1.97 ^#^	2.82 ^#^
17	Maltose	12.33 ^#^	0.75	0.83 ^#^
18	Uracil ^a^	0.40 ^#^	1.75 ^#^	2.81 ^#^
19	Hypoxanthine	0.33 ^#^	1.18	1.53 ^#^
20	LysoPC 22:6	4.12 ^#^	0.51 ^#^	0.58 ^#^
21	LysoPC 20:4	5.88 ^#^	0.50 ^#^	0.48 ^#^
22	LysoPC 22:6	1.72 ^#^	0.86 ^#^	0.78 ^#^
23	LysoPE 18:2 ^a^	0.21 ^#^	1.11	2.25 ^#^
24	LysoPC 20:4	2.50 ^#^	0.79 ^#^	0.67 ^#^
25	LysoPC 18:2 ^a^	0.50 ^#^	1.01	1.53 ^#^
26	LysoPC 18:2	0.45 ^#^	1.25	2.32 ^#^
27a	LysoPC 16:0 ^a^	0.64 ^#^	1.70 ^#^	1.39 ^#^
27b	LysoPC 20:3	3.44 ^#^	0.79	0.64 ^#^
28	LysoPE 18:0	0.23 ^#^	5.76 ^#^	3.06 ^#^
29	PE 36:3	0.51 ^#^	0.89	1.78 ^#^
30	PE 36:2	0.42 ^#^	0.90	2.22 ^#^
31	PC 38:9	1.79 ^#^	0.92	0.60 ^#^
32	TG 50:2	2.52 ^#^	0.78	0.45 ^#^

ND, group fed with a normal diet; HFD, group fed with a high-fat diet; HPGL, group fed with a high-fat diet and 1% *P. grandiflorum*; HPGH, group fed with a high-fat diet and 5% *P. grandiflorum*. ^1^ Fold change was calculated by dividing the mean of the peak intensity of each metabolite from each of the two groups. ^a^ Metabolites were only selected in DM extracts of liver tissues. ^#^ Metabolites showing significant differences (*p* < 0.05) between groups as determined by Student’s *t*-test. LysoPC 18:2 was selected in both UPLC-Q-TOF-MS analysis (No. 25a) and direct infusion MS analysis (No. 25b).

## Supplementary Materials

The following are available online at http://www.mdpi.com/2072-6643/9/1/071/s1, Figure S1: Effects of dietary *Platycodon grandiflorum* on body weight in high-fat diet (HFD)-fed C57BL/6J mice. The data are the mean ± SEM. Figure S2: PCA (A,C) and 3D PLS-DA (B,D) score plots derived from GC-TOF-MS and direct infusion nanoelectrospray-MS data sets for methanol extracts of serum in mice fed a High-Fat Diet (HFD) including *Platycodon grandiflorum*. Figure S3: PCA score plots derived from GC-TOF-MS (A,C), UPLC-Q-TOF-MS (B,D) data sets for MW (A,B) and DM (C,D) extracts, and direct infusion nanoelectrospray-MS data set (E) of liver in mice fed a High-Fat Diet (HFD) including *Platycodon grandiflorum*. Table S1: List of significantly discriminated serum metabolites obtained from GC-TOF-MS data sets for MW and DM extracts of liver tissues in mice fed a High-Fat Diet (HFD) including *Platycodon grandiflorum*. Table S2: List of significantly discriminated serum metabolites obtained from direct infusion nanoelectrospray-MS data sets in mice fed a High-Fat Diet (HFD) including *Platycodon grandiflorum*.
