# Supplementary material for: Mass Spectrometry-Based Metabolomic and Lipidomic Analyses of the Effects of Dietary Platycodon grandiflorum on Liver and Serum of Obese Mice under a High-Fat Diet
Source: Nutrients. 2017 Jan 17;9(1):71. doi: 10.3390/nu9010071 (PMC5295115; doi:10.3390/nu9010071)
Supplement: Supplementary file 1 [file nutrients-09-00071-s001.docx]

**Supplementary Materials: Mass Spectrometry-Based Metabolomic and Lipidomic Analyses of the Effects of Dietary *Platycodon grandiflorum* on Liver and Serum of Obese Mice under a High-Fat Diet**

Hye Min Park, Kab-Tae Park, Edmond Changkyun Park, Seungil Kim, Myung Sook Choi,
Kwang-Hyeon Liu and Choong Hwan Lee


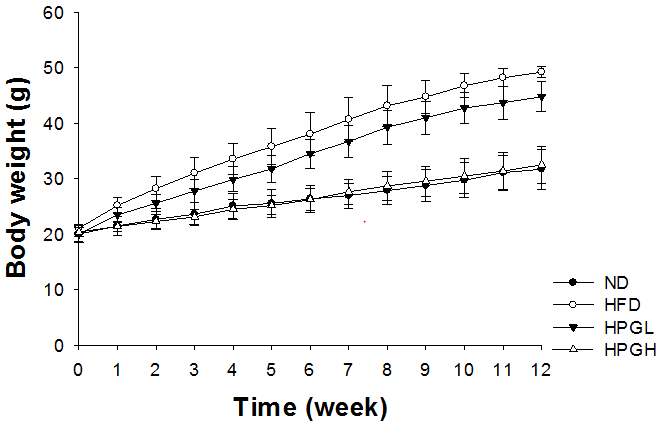


**Figure S1.** Effects of dietary *Platycodon grandiflorum* on body weight in a high-fat diet (HFD)-fed C57BL/6J mice. The data are the mean ± standard error of the mean (SEM).


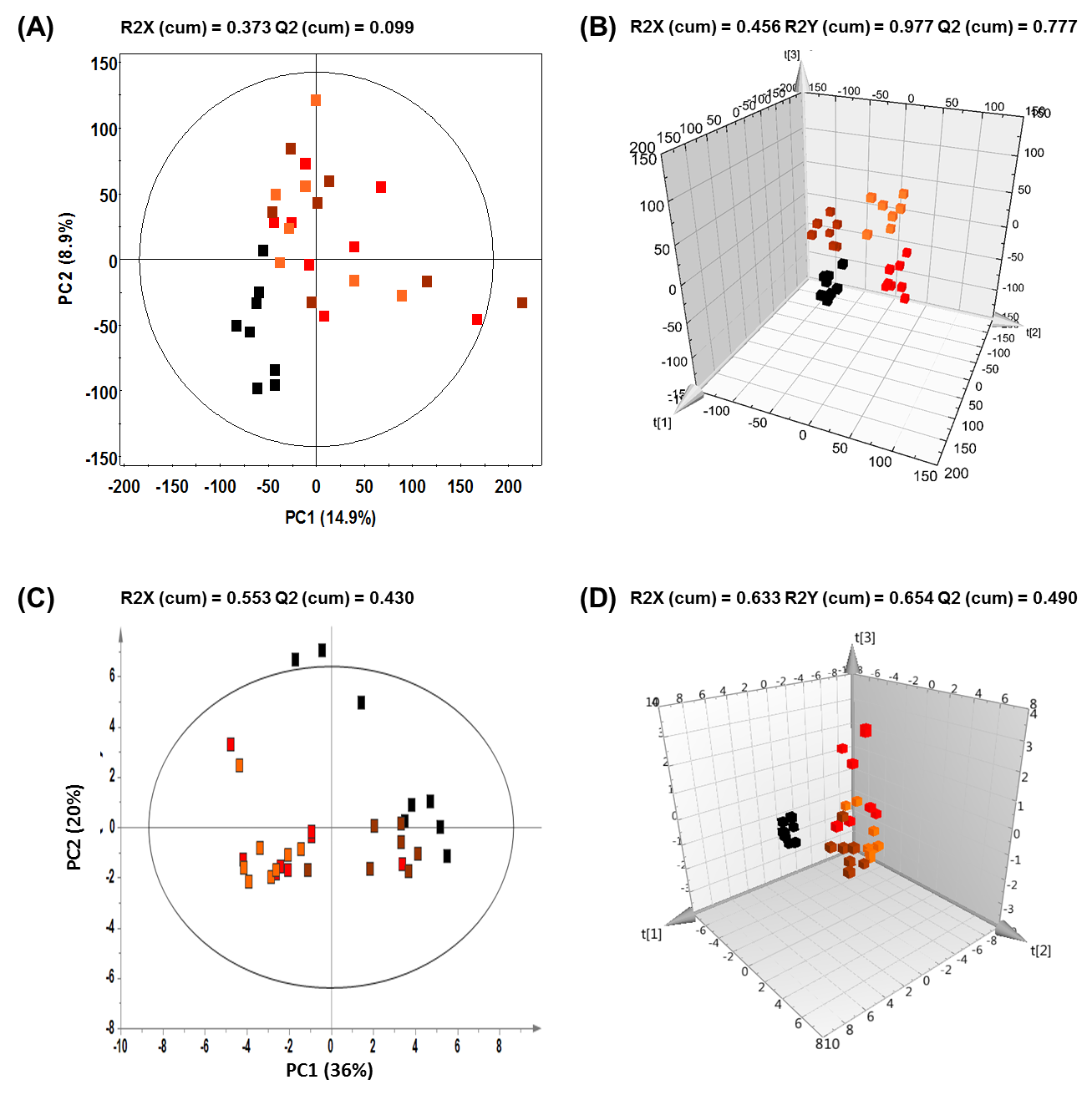


**Figure S2.** PCA (**A**,**C**) and 3D PLS-DA (**B**,**D**) score plots derived from GC-TOF-MS and direct infusion nanoelectrospray-MS data sets for methanol extracts of serum in mice fed a High-Fat Diet (HFD) including *Platycodon grandiflorum.* Black square ND, normal diet; red square HFD, high-fat diet; orange square HPGL, high-fat diet with 1% *P. grandiflorum*; brown square HPGH, high-fat diet with 5% *P. grandiflorum*.


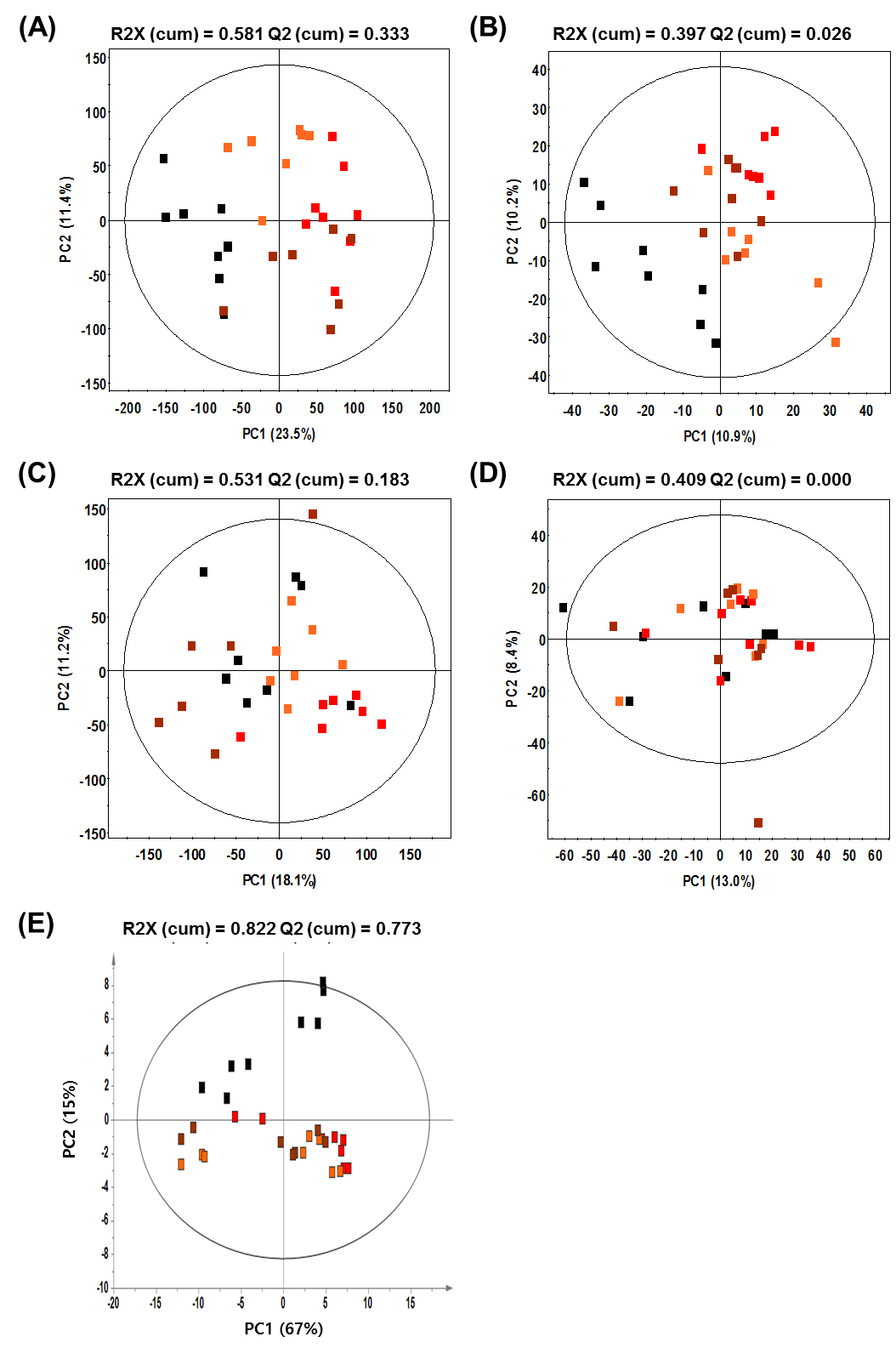


**Figure S3.** PCA Score Plots Derived from GC-TOF-MS (**A**,**C**), UPLC-Q-TOF-MS (**B**,**D**) data sets for MW (**A**,**B**) and DM (**C**,**D**) extracts, and direct infusion nanoelectrospray-MS data set (**E**) of liver in mice fed a High-Fat Diet (HFD) including *Platycodon grandiflorum*. Black square ND, normal diet; red square HFD, high-fat diet; orange square HPGL, high-fat diet with 1% *P. grandiflorum*; brown square HPGH, high-fat diet with 5% *P. grandiflorum*.

**Table S1.** List of significantly discriminated serum metabolites obtained from GC-TOF-MS data sets for MW and DM extracts of liver tissues in mice fed a High-Fat Diet (HFD) including *Platycodon grandiflorum*.

| **t_R_ (min) ^1^** | **Unique *m/z*** | **Metabolite** | **Derivatized** | **Fold Change ^2^** | | | **ID ^3^** |
| --- | --- | --- | --- | --- | --- | --- | --- |
|  |  |  |  | **HFD/ND** | **HPGL/HFD** | **HPGH/HFD** |  |
| *Amino acids* | | | | | | | |
| 9.37 | 176 | Methionine | (TMS) ^2^ | 0.99 | 0.73 ^#^ | 1.31 | STD/MS ^4^ |
| 0.15 | 246 | Glutamic acid | (TMS) ^3^ | 1.53 ^#^ | 0.75 ^#^ | 0.73 | STD/MS |
| 11.64 | 142 | Ornithine | (TMS) ^4^ | 1.38 ^#^ | 0.76 ^#^ | 0.72 ^#^ | STD/MS |
| 12.37 | 156 | Lysine | (TMS) ^4^ | 0.76 ^#^ | 0.74 ^#^ | 1.18 | STD/MS |
| 14.27 | 202 | Tryptophan | (TMS) ^3^ | 1.26 ^#^ | 0.85 ^#^ | 0.78 ^#^ | STD/MS |
| 14.67 | 218 | Cystine | (TMS) ^4^ | 0.93 | 0.80 | 0.49 ^#^ | MS |
| *Organic compounds* | | | | | | | |
| 6.03 | 117 | Hydroxybutyric acid | (TMS) ^2^ | 0.34 ^#^ | 1.93 | 1.16 | MS |
| 6.09 | 145 | Hydroxyisovaleric acid | (TMS) ^2^ | 0.67 ^#^ | 0.98 | 0.78 | MS |
| 7.95 | 117 | Dihydroxybutanoic acid | (TMS) ^3^ | 0.75 ^#^ | 1.12 | 0.77 | MS |
| *Carbohydrates* | | | | | | | |
| 11.92 | 217 | Saccharide * |  | 0.63 ^#^ | 0.88 ^#^ | 0.75 ^#^ | MS |
| 12.44 | 103 | Saccharide * |  | 1.18 ^#^ | 1.06 | 1.00 | MS |
| 12.54 | 103 | Sorbitol | (TMS) ^6^ | 2.60 ^#^ | 0.75 | 0.72 | STD/MS |
| 12.65 | 217 | Saccharide * |  | 1.67 ^#^ | 0.92 | 0.79 ^#^ | MS |
| 12.83 | 103 | Saccharide * |  | 3.11 ^#^ | 0.92 | 0.87 | MS |
| 12.88 | 217 | Saccharide * |  | 2.53 ^#^ | 1.00 | 0.84 | MS |
| 13.11 | 204 | Saccharide * |  | 1.13 ^#^ | 1.05 | 0.99 | MS |
| 13.50 | 204 | Saccharide * |  | 1.68 ^#^ | 1.01 | 1.04 | MS |
| *Fatty acids and Lipid* | | | | | | | |
| 11.75 | 117 | Myristic acid | TMS | 0.41 ^#^ | 1.38 ^#^ | 1.60 | STD/MS |
| 12.93 | 117 | Palmitoleic acid | TMS | 0.50 ^#^ | 1.25 | 1.19 | STD/MS |
| 14.09 | 337 | Linoleic acid | TMS | 0.37 ^#^ | 0.87 | 1.17 | STD/MS |
| 16.01 | 91 | Docosahexaenoic acid | TMS | 0.70 ^#^ | 1.08 | 0.89 | STD/MS |
| 19.61 | 129 | Cholesterol | TMS | 0.84 | 0.94 | 0.83 ^#^ | STD/MS |

Variables were determined by using the VIP value (>1.0) and *p*-value (<0.05) from the PLS-DA model. ND, group fed with a normal diet; HFD, group fed with a high-fat diet; HPGL, group fed with a high-fat diet and 1% *P. grandiflorum*; HPGH, group fed with a high-fat diet and 5% *P. grandiflorum.* ^1^ t_R_ was the retention time. ^2^ Fold change was calculated by dividing the mean of the peak intensity of each metabolite from each of the two groups. ^3^ ID, identification. ^4^ Metabolites were identified using commercial standard compounds (STD) in comparison with the mass spectra (MS) and retention time. * Saccharide was not successfully identified, but its mass fragments were similar to general mass fragments of saccharides. ^#^ Metabolites showing significant differences (*p <* 0.05) between groups as determined by Student’s *t*-test

**Table S2.** List of significantly discriminated serum metabolites obtained from direct infusion nanoelectrospray-MS data sets in mice fed a High-Fat Diet (HFD) including *Platycodon grandiflorum*.

| **No.** | **Identifiedion (*m/z*)** | **Metabolite ^1^** | **Adduct** | **Fold Change ^2^** | | |
| --- | --- | --- | --- | --- | --- | --- |
|  |  |  |  | **HFD/ND** | **HPGL/HFD** | **HPGH/HFD** |
| 1 | 520.5 | LysoPC 18:2 | M + H^+^ | 0.67 ^#^ | 0.93 | 1.19 |
| 2 | 544.5 | LysoPC 20:4 | M + H^+^ | 1.65 ^#^ | 0.98 | 0.66 ^#^ |
| 3 | 690.3 | CE 20:4 | M + NH_4_^+^ | 2.25 ^#^ | 1.45 ^#^ | 0.88 |
| 4 | 692.5 | CE 20:3 | M + NH_4_^+^ | 2.08 ^#^ | 1.43 ^#^ | 0.85 |
| 5 | 756.7 | PC 32:0 | M + Na^+^ | 0.562 ^#^ | 0.94 | 1.01 |
| 6 | 758.7 | PC 34:2 (16:0/18:2) | M + H^+^ | 0.55 ^#^ | 0.88 | 1.39 ^#^ |
| 7 | 780.7 | PC 34:2 | M + Na^+^ | 0.48 ^#^ | 0.99 | 1.24 |
| 8 | 782.7 | PC 36:4 (16:0/20:4) | M + H^+^ | 1.50 ^#^ | 1.11 | 0.71 ^#^ |
| 9 | 784.7 | PC 36:3 (16:0/20:3) | M + H^+^ | 1.20 ^#^ | 1.06 | 0.96 |
| 10 | 808.7 | PC 36:2 | M + Na^+^ | 1.28 ^#^ | 1.11 | 0.85 |
| 11 | 810.7 | PC 38:4 (18:0/20:4) | M + H^+^ | 2.28 ^#^ | 1.01 | 0.60 ^#^ |
| 12 | 812.7 | PC 38:3 (18:0/20:3) | M + H^+^ | 2.07 ^#^ | 1.00 | 0.65 ^#^ |
| 13 | 834.7 | PC 38:3 | M + Na^+^ | 1.29 ^#^ | 1.07 | 0.77 ^#^ |
| 14 | 846.6 | TG 50:3 | M + NH_4_^+^ | 0.53 ^#^ | 0.88 | 1.52 |
| 15 | 868.6 | TG 52:6 | M + NH_4_^+^ | 0.55 ^#^ | 0.82 | 0.80 |
| 16 | 870.6 | TG 52:5 | M + NH_4_^+^ | 0.25 ^#^ | 0.76 | 1.47 |
| 17 | 872.6 | TG 52:4 | M + NH_4_^+^ | 0.26 ^#^ | 0.61 | 1.84 |
| 18 | 874.6 | TG 52:3 | M + NH_4_^+^ | 0.53 ^#^ | 0.66 | 2.06 ^#^ |
| 19 | 894.6 | TG 54:7 | M + NH_4_^+^ | 0.32 ^#^ | 0.86 | 1.18 |
| 20 | 896.6 | TG 54:6 | M + NH_4_^+^ | 0.36 ^#^ | 0.66 | 1.40 |
| 21 | 898.7 | TG 54:5 | M + NH_4_^+^ | 0.42 ^#^ | 0.67 | 1.90 |
| 22 | 918.6 | TG56:9 | M + NH_4_^+^ | 0.46 ^#^ | 1.03 | 1.07 |
| 23 | 920.6 | TG 56:8 | M + NH_4_^+^ | 0.41 ^#^ | 0.75 | 1.14 |
| 24 | 922.7 | TG 56:7 | M + NH_4_^+^ | 0.62 | 0.74 | 1.29 |
| 25 | 944.6 | TG 58:10 | M + NH_4_^+^ | 0.59 ^#^ | 0.88 | 0.83 |

^1^ Variables were selected on the basis of VIP value (>0.7) and *p*-value (<0.05) upon PLS-DA modeling. ^2^ Fold change was calculated by dividing the mean of the peak intensity of each metabolite from each of the two groups. LysoPC; lysophosphatidylcholine, PC; phosphatidylcholine, PE; phosphatidylethanolamine, TG; triacylglycerol. ^#^ Metabolites showing significant differences (*p <* 0.05) between groups as determined by Student’s *t*-test.

**Table S3.** List of significantly discriminated hepatic metabolites obtained from GC-TOF-MS data sets for MW and DM extracts of liver tissues in mice fed a High-Fat Diet (HFD) including *Platycodon grandiflorum*.

| **t_R_ (min) ^1^** | **Unique *m/z*** | **Metabolite** | **Derivatized** | **Fold Change ^2^** | | | **ID ^3^** |
| --- | --- | --- | --- | --- | --- | --- | --- |
|  |  |  |  | **HFD/ND** | **HPGL/HFD** | **HPGH/HFD** |  |
| *Amino acids* | | | | | | | |
| 5.37 | 116 | Alanine | (TMS)_2_ | 0.60 ^#^ | 1.04 | 1.03 | STD/MS ^4^ |
| 6.56 | 144 | Valine | (TMS)_2_ | 0.65 ^#^ | 1.18 | 1.21 | STD/MS |
| 7.33 | 158 | Isoleucine | (TMS)_2_ | 0.62 ^#^ | 1.32 | 1.27 | STD/MS |
| 7.38 | 142 | Proline | (TMS)_2_ | 0.49 ^#^ | 1.26 | 1.43 | STD/MS |
| 7.46 | 174 | Glycine | (TMS)_3_ | 0.57 ^#^ | 1.27 ^#^ | 1.18 ^#^ | STD/MS |
| 7.97 | 204 | Serine | (TMS)_3_ | 0.50 ^#^ | 1.21 | 1.34 ^#^ | STD/MS |
| 8.22 | 219 | Threonine | (TMS)_3_ | 0.51 ^#^ | 1.25 | 1.37 ^#^ | STD/MS |
| 9.36 | 232 | Aspartic acid | (TMS)_3_ | 0.49 ^#^ | 1.09 | 1.57 | STD/MS |
| 9.36 | 176 | Methionine ^a^ | (TMS)_2_ | 0.31 ^#^ | 1.25 | 2.38 ^#^ | STD/MS |
| 9.41 | 156 | Pyroglutamic acid | (TMS)_2_ | 0.71 ^#^ | 1.26 ^#^ | 1.14 | STD/MS |
| 10.14 | 246 | Glutamic acid | (TMS)_3_ | 0.45 ^#^ | 1.42 | 1.71 ^#^ | STD/MS |
| 10.24 | 218 | Phenylalanine ^a^ | (TMS)_2_ | 0.39 ^#^ | 1.19 | 2.40 ^#^ | STD/MS |
| 11.62 | 142 | Ornithine | (TMS)_4_ | 0.44 ^#^ | 1.26 | 1.67 ^#^ | STD/MS |
| 12.33 | 156 | Lysine ^a^ | (TMS)_4_ | 0.39 ^#^ | 1.63 ^#^ | 2.98 ^#^ | STD/MS |
| 12.46 | 218 | Tyrosine ^a^ | (TMS)_3_ | 0.42 ^#^ | 1.24 | 2.47 ^#^ | STD/MS |
| *Organic compounds* | | | | | | | |
| 4.92 | 117 | Lactate | (TMS)_2_ | 0.87 ^#^ | 1.26 ^#^ | 0.89 | STD/MS |
| 5.96 | 117 | Hydroxybutyric acid | (TMS)_2_ | 0.38 ^#^ | 2.19 ^#^ | 1.02 | MS |
| 6.89 | 189 | Urea | (TMS)_2_ | 0.71 ^#^ | 1.15 | 1.15 ^#^ | STD/MS |
| 7.47 | 247 | Succinic acid | (TMS)_2_ | 0.42 ^#^ | 1.11 | 1.75 ^#^ | STD/MS |
| 7.80 | 245 | Fumaric acid ^a^ | (TMS)_2_ | 0.34 ^#^ | 1.67 | 2.96 ^#^ | STD/MS |
| 9.09 | 133 | Malonic acid ^a^ | (TMS)_3_ | 0.45 ^#^ | 1.40 | 2.27 | STD/MS |
| 10.59 | 326 | Taurine | (TMS)_3_ | 0.20 ^#^ | 1.45 ^#^ | 1.11 | STD/MS |
| 12.12 | 103 | D-Gluconic acid deriv. ^a^ |  | 1.12 | 1.31 | 1.50 ^#^ | STD/MS |
| 13.06 | 103 | Gluconic acid | (TMS)_5_ | 0.64 ^#^ | 0.95 | 1.23 | STD/MS |
| *Fatty acids and Lipids* | | | | | | | |
| 13.04 | 313 | Palmitic acid ^a^ | TMS | 1.02 | 0.94 | 0.78 ^#^ | STD/MS |
| 13.33 | 122 | Oleanitrile ^a^ |  | 1.12 | 0.87 | 0.73 ^#^ | MS |
| 13.38 | 67 | Linolelidic acid methyl ester ^a^ |  | 1.53 ^#^ | 0.85 ^#^ | 0.54 ^#^ | MS |
| 13.41 | 55 | Oleic acid methyl ester ^a^ |  | 2.45 ^#^ | 1.56 ^#^ | 0.54 ^#^ | MS |
| 13.97 | 75 | γ-Linolenic acid ^a^ | TMS | 0.20 ^#^ | 1.41 | 2.76 | STD/MS |
| 14.07 | 337 | Linoleic acid ^a^ | TMS | 0.28 ^#^ | 1.34 | 1.89 | STD/MS |
| 14.09 | 339 | Oleic acid | TMS | 0.40 ^#^ | 2.48 ^#^ | 1.48 | STD/MS |
| 14.22 | 341 | Stearic acid | TMS | 0.67 ^#^ | 1.51 ^#^ | 1.09 | STD/MS |
| 14.39 | 79 | Arachidonic acid methyl ester ^a^ |  | 1.71 ^#^ | 1.35 | 0.66 | MS |
| 15.03 | 91 | Eicosapentaenoic acid ^a^ | TMS | 0.45 ^#^ | 1.20 | 0.93 | STD/MS |
| 15.24 | 131 | Oleamide ^a^ | (TMS)_2_ | 1.21 ^#^ | 0.90 | 0.72 ^#^ | STD/MS |
| 16.00 | 91 | Docosahexaenoic acid ^a^ | TMS | 0.65 ^#^ | 1.44 ^#^ | 1.16 | STD/MS |
| 19.57 | 129 | Cholesterol | TMS | 0.24 ^#^ | 1.97 ^#^ | 2.82 ^#^ | STD/MS |
| *Carbohydrates including sugar and sugar alcohols* | | | | | | | |
| 7.14 | 205 | Glycerol | (TMS)_3_ | 0.77 ^#^ | 1.30 ^#^ | 0.81 | STD/MS |
| 10.46 | 204 | Saccharide * |  | 0.63 ^#^ | 1.24 | 0.98 | MS |
| 11.01 | 103 | Arabitol | (TMS)_5_ | 0.49 ^#^ | 1.29 | 1.26 | STD/MS |
| 12.13 | 204 | Saccharide * |  | 6.96 ^#^ | 0.55 ^#^ | 1.08 | MS |
| 12.19 | 160 | Mannose | MeOX, (TMS)_5_ | 2.23^#^ | 0.59 ^#^ | 1.43 | STD/MS |
| 12.28 | 205 | Glucose | MeOX, (TMS)_5_ | 1.02 | 1.08 | 0.81 ^#^ | STD/MS |
| 12.41 | 103 | Glucose deriv. |  | 1.07 | 1.28 ^#^ | 0.93 | STD/MS |
| 12.53 | 103 | Sorbitol | (TMS)_6_ | 3.56 ^#^ | 0.37 ^#^ | 1.29 | STD/MS |
| 12.64 | 217 | Saccharide * |  | 3.28 | 0.45 ^#^ | 0.75 | MS |
| 12.73 | 103 | Galacturonic acid deriv. ^a^ |  | 0.74 | 1.27 | 3.04 ^#^ | STD/MS |
| 13.10 | 204 | Saccharide * |  | 1.98 ^#^ | 0.91 | 0.91 | MS |
| 13.52 | 217 | *myo*-Inositol | (TMS)_6_ | 0.43 ^#^ | 1.45 ^#^ | 1.30 | STD/MS |
| 13.83 | 205 | Saccharide * |  | 1.13 | 0.80 | 1.55 ^#^ | MS |
| 16.77 | 204 | Saccharide * |  | 19.06 ^#^ | 0.27 ^#^ | 1.05 | MS |
| 16.96 | 204 | Lactose | MeOX, (TMS)_8_ | 19.19 ^#^ | 0.28 ^#^ | 0.99 | STD/MS |
| 17.12 | 204 | Maltose | MeOX, (TMS)_8_ | 12.33 ^#^ | 0.75 | 0.83 ^#^ | STD/MS |
| 17.26 | 204 | Maltose deriv. |  | 27.68 ^#^ | 0.40 ^#^ | 0.83 | STD/MS |
| 17.37 | 103 | Saccharide* |  | 5.29 ^#^ | 0.56 ^#^ | 0.96 | MS |
| *Nucleobases* | | | | | | | |
| 7.79 | 99 | Uracil ^a^ | (TMS)_2_ | 0.40 ^#^ | 1.75 ^#^ | 2.81 ^#^ | STD/MS |
| 11.58 | 265 | Hypoxanthine | (TMS)_2_ | 0.33 ^#^ | 1.18 | 1.53 ^#^ | STD/MS |
| 15.55 | 217 | Uridine | (TMS)_3_ | 0.56 ^#^ | 1.00 | 1.27 | STD/MS |
| 16.17 | 217 | Inosine ^a^ | (TMS)_4_ | 0.57 | 1.89 ^#^ | 1.90 ^#^ | STD/MS |
| *Inorganic compounds* | | | | | | | |
| 7.17 | 299 | Phosphoric acid | (TMS)_3_ | 0.95 | 1.08 | 0.78 ^#^ | STD/MS |
| 10.56 | 451 | Pyrophosphate ^a^ | (TMS)_4_ | 1.21 | 0.76 ^#^ | 0.65 ^#^ | MS |

Variables were determined by using the VIP value (>1.0) and *p*-value (<0.05) from the PLS-DA model. ND, group fed with a normal diet; HFD, group fed with a high-fat diet; HPGL, group fed with a high-fat diet and 1% *P. grandiflorum*; HPGH, group fed with a high-fat diet and 5% *P. grandiflorum.* ^1^ t_R_ was the retention time. ^2^ Fold change was calculated by dividing the mean of the peak intensity of each metabolite from each of the two groups. ^3^ ID, identification. ^4^ Metabolites were identified using commercial standard compounds (STD) in comparison with the mass spectra (MS) and retention time. * Saccharide was not successfully identified, but its mass fragments were similar to general mass fragments of saccharides. ^a^ Metabolites were only selected in DM extracts of liver tissues. ^#^ Metabolites showing significant differences (*p <* 0.05) between groups as determined by Student’s *t*-test.

**Table S4.** List of significantly discriminated hepatic metabolites obtained from UPLC-Q-TOF-MS data sets for MW and DM extracts of liver tissues in mice fed a High-Fat Diet including *Platycodon grandiflorum*.

| **t_R_ (min) ^1^** | **Metabolite ^2^** | **Measured *m/z*** | **Calculated *m/z*** | **mDa** | **DBE** | **iFit (norm)** | **Elemental Composition** | **Adduct** | **Fold Change ^3^** | | |
| --- | --- | --- | --- | --- | --- | --- | --- | --- | --- | --- | --- |
|  |  |  |  |  |  |  |  |  | **HFD/ND** | **HPGL/HFD** | **HPGH/HFD** |
| *Amino acids* | | | | | | | | | | | |
| 0.75 | Lysine | 145.0963 | 145.0977 | −1.4 | 1.5 | 1.8 | C6 H13 N2 O2 | M-H | 0.57 ^#^ | 0.72 | 1.23 |
| 0.77 | Histidine | 154.0603 | 154.0617 | −1.4 | 4.5 | 2.2 | C6 H8 N3 O2 | M-H | 0.61 ^#^ | 0.78 | 0.99 |
| 1.44 | xanthine ^a^ | 151.0232 | 151.0256 | −2.4 | 6.5 | 1.9 | C5 H3 N4 O2 | M-H | 0.69 | 1.34 | 2.32 ^#^ |
| *Bile acids* | | | | | | | | | | | |
| 5.91 | Bile acids * | 407.2802 | 407.2797 | 0.5 | 5.5 | 0.5 | C24 H39 O5 | M-H | 0.48 ^#^ | 0.82 | 1.29 |
| 6.06 | Bile acids * | 407.2780 | 407.2797 | −1.7 | 5.5 | 0.1 | C24 H39 O5 | M-H | 0.41 ^#^ | 0.77 | 0.91 |
| *Glycerophospholipids* ^δ^ | | | | | | | | | | | |
| 7.92 | LysoPC 16:1 | 478.2955 | 478.2934 | 2.1 | 2.5 | 2.7 | C23 H45 N O7 P | M-CH_3_ | 0.69 ^#^ | 1.06 | 0.82 |
| 8.02 | LysoPC 22:6 | 552.3113 | 552.3090 | 2.3 | 7.5 | 1.8 | C29 H47 N O7 P | M-CH_3_ | 4.12 ^#^ | 0.51 ^#^ | 0.58 ^#^ |
| 8.04 | LysoPE 18:2 ^a^ | 476.2774 | 476.2777 | −0.3 | 3.5 | 3.5 | C23 H43 N O7 P | M-H | 0.33 ^#^ | 0.78 | 1.33 |
| 8.05 | LysoPC 18:2 ^a^ | 504.3113 | 504.3090 | 2.3 | 3.5 | 1.9 | C25 H47 N O7 P | M-CH_3_ | 0.62 ^#^ | 0.75 ^#^ | 1.12 |
| 8.07 | LysoPC 20:4 | 528.3096 | 528.3090 | 0.6 | 5.5 | 1.8 | C27 H47 N O7 P | M-CH_3_ | 5.88 ^#^ | 0.50 ^#^ | 0.48 ^#^ |
| 8.13 | LysoPE 22:6 | 524.2783 | 524.2777 | 0.6 | 7.5 | 0.9 | C27 H43 N O7 P | M-H | 0.68 ^#^ | 1.44 ^#^ | 0.95 |
| 8.15 | LysoPC 22:6 | 552.3093 | 552.3090 | 0.3 | 7.5 | 0.6 | C29 H47 N O7 P | M-CH_3_ | 1.72 ^#^ | 0.86 ^#^ | 0.78 ^#^ |
| 8.19 | LysoPE 18:2 ^a^ | 476.2781 | 476.2777 | 0.4 | 3.5 | 3.6 | C23 H43 N O7 P | M-H | 0.21 ^#^ | 1.11 | 2.25 ^#^ |
| 8.21 | LysoPC 20:4 | 528.3087 | 528.3090 | −0.3 | 5.5 | 0.3 | C27 H47 N O7 P | M-CH_3_ | 2.50 ^#^ | 0.79 ^#^ | 0.67 ^#^ |
| 8.23 | LysoPC 18:2 ^a^ | 504.3107 | 504.3090 | 1.7 | 3.5 | 0.4 | C25 H47 N O7 P | M-CH_3_ | 0.50 ^#^ | 1.01 | 1.53 ^#^ |
| 8.41 | LysoPC 20:3 ^a^ | 530.3255 | 530.3247 | 0.8 | 4.5 | 1.9 | C27 H49 N O7 P | M-CH_3_ | 2.64 ^#^ | 1.00 | 0.86 |
| 8.46 | LysoPC 16:0 ^a^ | 480.3082 | 480.309 | −0.8 | 1.5 | 4.2 | C23 H47 N O7 P | M-CH_3_ | 0.64 ^#^ | 1.70 ^#^ | 1.39 ^#^ |
| 8.58 | LysoPC 20:3 | 530.3294 | 530.3247 | 4.7 | 4.5 | 3.7 | C27 H49 N O7 P | M-CH_3_ | 3.44 ^#^ | 0.79 | 0.64 ^#^ |
| 8.68 | LysoPE 16:0 ^a^ | 452.2701 | 452.2777 | −7.6 | 1.5 | 0.6 | C21 H43 N O7 P | M-H | 0.49 ^#^ | 1.37 | 1.16 |
| 8.72 | LysoPC 18:1 | 506.3257 | 506.3247 | 1.0 | 2.5 | 5.5 | C25 H49 N O7 P | M-CH_3_ | 4.19 | 0.50 ^#^ | 0.77 |
| 8.73 | LysoPC 16:0 ^a^ | 480.3040 | 480.3090 | −5.0 | 1.5 | 1.1 | C23 H47 N O7 P | M-CH_3_ | 1.09 | 0.90 | 0.80 ^#^ |
| 8.88 | LysoPE 18:1 | 478.2971 | 478.2934 | 3.7 | 2.5 | 3.7 | C23 H45 N O7 P | M-H | 0.61 | 2.49 ^#^ | 1.47 |
| 8.91 | LysoPC 18:1 | 506.3254 | 506.3247 | 0.7 | 2.5 | 4.7 | C25 H49 N O7 P | M-CH_3_ | 1.66 ^#^ | 1.02 | 0.96 |
| 9.74 | LysoPE 18:0 | 480.3110 | 480.3090 | 2.0 | 1.5 | 2.8 | C23 H47 N O7 P | M-H | 0.23 ^#^ | 5.76 ^#^ | 3.06 ^#^ |
| *Other* | | | | | | | | | | | |
| 4.26 | Prostaglandins ^ζ^ | 353.2344 | 353.2328 | 1.6 | 4.5 | 2.5 | C20 H33 O5 | M-H | 0.42 ^#^ | 0.77 | 1.31 |

Variables were determined by using the VIP value (>1.0) and *p*-value (<0.05) from the PLS-DA model. ND, group fed with a normal diet; HFD, group fed with a high-fat diet; HPGL, group fed with a high-fat diet and 1% *P. grandiflorum*; HPGH, group fed with a high-fat diet and 5% *P. grandiflorum.* LysoPC, lysophosphatidylcholine; LysoPE, lysophosphatidylethanolamine. ^1^ t_R_ was the retention time. ^2^ Assignment of metabolites contributing to the observed variance was performed by elemental composition analysis software with calculated mass, mass tolerance (mDa), double bond equivalents (DBEs), and the iFit algorithm as implemented in MassLynx software, and by using either commercial standard compounds compared with the retention time and mass spectra or the HMDB (The Human Metabolome Data Base (http://www.hmdb.ca/)). ^3^ Fold change was calculated by dividing the mean of the peak intensity of each metabolite from each of the two groups. ^a^ Metabolites were only selected in DM extract of liver tissue. ^*^ The class of primary bile acids including cholic acid and trihydroxy cholonic acid. ^δ^ The two forms of lysoPC and lysoPE, with the fatty acyl groups at positions 1 (sn-1) or 2 (sn-2) on the glycerol backbone. ^ζ^ The class of organic compounds known as prostaglandins and related compounds. ^#^ Metabolites showing significant differences (*p <* 0.05) between groups as determined by Student’s *t*-test.

**Table S5.** List of significantly discriminated hepatic metabolites obtained from direct infusion nanoelectrospray-MS data sets in mice fed a High-Fat Diet including *Platycodon grandiflorum*.

| **No.** | **Identified** | **Metabolite** | **Adduct** | **Fold Change ^1^** | | |
| --- | --- | --- | --- | --- | --- | --- |
|  | **ion (*m/z*)** |  |  | **HFD/ND** | **HPGL/HFD** | **HPGH/HFD** |
| 1 | 520.5 | LysoPC 18:2 | M + H^+^ | 0.45 ^#^ | 1.25 | 2.32 ^#^ |
| 2 | 716.5 | PE 34:2 | M + H^+^ | 0.32 ^#^ | 0.70 | 1.75 |
| 3 | 742.6 | PE 36:3 | M + H^+^ | 0.51 ^#^ | 0.89 | 1.78 ^#^ |
| 4 | 744.6 | PE 36:2 | M + H^+^ | 0.42 ^#^ | 0.90 | 2.22 ^#^ |
| 5 | 756.7 | PC 32:0 | M + Na^+^ | 0.27 ^#^ | 1.40 | 2.24 |
| 6 | 758.7 | PC 34:2 (16:0/18:2) | M + H^+^ | 0.28 ^#^ | 1.30 | 2.39 |
| 7 | 780.7 | PC 34:2 | M + Na^+^ | 0.24 ^#^ | 1.78 | 2.79 |
| 8 | 822.7 | PC 38:9 | M + Na^+^ | 1.79 ^#^ | 0.92 | 0.60 ^#^ |
| 9 | 844.6 | TG 50:4 | M + NH_4_^+^ | 0.69 ^#^ | 0.88 | 0.60 ^#^ |
| 10 | 848.6 | TG 50:2 | M + NH_4_^+^ | 2.52 ^#^ | 0.78 | 0.45 ^#^ |
| 11 | 850.7 | TG 50:1 | M + NH_4_^+^ | 3.91 ^#^ | 0.77 | 0.55 |
| 12 | 862.7 | TG 51:2 | M + NH_4_^+^ | 3.01 ^#^ | 0.80 | 0.82 |
| 13 | 868.6 | TG 52:6 | M + NH_4_^+^ | 0.34 ^#^ | 0.75 | 0.65 |
| 14 | 870.7 | TG 52:5 | M + NH_4_^+^ | 0.43 ^#^ | 0.66 | 0.59 |
| 15 | 872.7 | TG 52:4 | M + NH_4_^+^ | 0.65 | 0.63 | 0.62 |
| 16 | 876.7 | TG 52:2 | M + NH_4_^+^ | 3.58 ^#^ | 0.71 | 0.73 |
| 17 | 878.7 | TG 52:1 | M + NH_4_^+^ | 4.28 ^#^ | 0.71 | 0.78 |
| 18 | 894.6 | TG 54:7 | M + NH_4_^+^ | 0.35 ^#^ | 0.76 | 0.62 |
| 19 | 896.6 | TG 54:6 | M + NH_4_^+^ | 0.49 ^#^ | 0.63 | 0.65 |
| 20 | 898.7 | TG 54:5 | M + NH_4_^+^ | 0.71 | 0.60 | 0.68 |
| 21 | 902.7 | TG 54:3 | M + NH_4_^+^ | 3.00 ^#^ | 0.73 | 0.68 |
| 22 | 904.7 | TG 54:2 | M + NH_4_^+^ | 3.15 ^#^ | 0.78 | 0.85 |
| 23 | 906.8 | TG 54:1 | M + NH_4_^+^ | 2.58 ^#^ | 0.77 | 1.02 |
| 24 | 918.6 | TG 56:9 | M + NH_4_^+^ | 0.57 ^#^ | 0.76 | 0.66 |
| 25 | 920.6 | TG 56:8 | M + NH_4_^+^ | 0.50 ^#^ | 0.75 | 0.81 |
| 26 | 930.7 | TG 56:3 | M + NH_4_^+^ | 2.91 ^#^ | 0.73 | 0.82 |
| 27 | 944.6 | TG 58:10 | M + NH_4_^+^ | 0.43 ^#^ | 0.69 | 0.80 |
| 28 | 946.6 | TG 58:9 | M + NH_4_^+^ | 0.49 ^#^ | 0.74 | 0.88 |

Variables were determined by using the VIP value (>0.7) and *p*-value (<0.05) from the PLS-DA model. ND, group fed with a normal diet; HFD, group fed with a high-fat diet; HPGL, group fed with a high-fat diet and 1% *P. grandiflorum*; HPGH, group fed with a high-fat diet and 5% *P. grandiflorum.* ^1^ Fold change was calculated by dividing the mean of the peak intensity of each metabolite from each of the two groups. LysoPC, lysophosphatidylcholine; PC, phosphatidylcholine; PE, phosphatidylethanolamine; TG, triacylglycerol. ^#^ Metabolites showing significant differences (*p <* 0.05) between groups as determined by Student’s *t*-test.
